# High efficacy of intravesical treatment of metformin on bladder cancer in preclinical model

**DOI:** 10.18632/oncotarget.6933

**Published:** 2016-01-18

**Authors:** Mei Peng, Qiongli Su, Qing Zeng, Le Li, Zhihong Liu, Lei Xue, Yan Cheng, Yanjun Huang, Ting Tao, Hongwei Lv, Xiaohui Li, Xiaojun Tao, Peng Guo, Alex F. Chen, Xiaoping Yang

**Affiliations:** ^1^ Department of Pharmacy, School of Medicine, Hunan Normal University, Changsha, Hunan, P.R. China 410013; ^2^ Department of Urology Surgery, Third Affiliated Hospital, Central South University, Changsha, Hunan, P.R. China 410023; ^3^ Third Affiliated Hospital and the Institute of Vascular Disease and Translational Medicine, Central South University, Changsha, Hunan, P.R. China 410023; ^4^ Department of Pathology, Hunan Provincial Cancer Hospital, Changsha, Hunan, P.R. China 410023; ^5^ School of Pharmacy, Central South University, Changsha, Hunan, P.R. China 410023; ^6^ Institute of Urology, Xi'an Jiaotong University, Xi'an, Shaanxi, P.R. China 710061

**Keywords:** metformin, bladder cancer, localized administration, AMPK, preclinical model

## Abstract

Anticancer potential of metformin has been extensively studied. However, its anticancer clinical use remains yet to be approved since sufficient concentration on target organs could not be achieved via conventional administration. To overcome this drawback, we aim to examine the efficiency of novel intravesical treatment of metformin on syngeneic orthotopic preclinical model. Three human and one murine bladder cancer cell lines were tested *in vitro* for inhibitory sensitivity by MTT and cologenic assays. AMPK pathway including AKT, Erk and S6K was examined by western blot and further explored by regulating activated levels using specific inhibitors. *In vivo* efficacy was determined by Kaplan-Meier survival curves and measurements of body and bladder weights plus tumor biomarkers. Lactic acid and metformin levels of plasma were measured by standard procedures. The results demonstrated that metformin activated AMPK and decreased phosphorylation of Akt and Erk. Furthermore, combinations of metformin with either Akt or Erk inhibitors synergistically diminished cancer proliferation, suggesting the involvement of Akt- and Erk- related pathways. Intravesical metformin 26 and 104 mg/kg, twice per week demonstrated a rapid elimination of the implanted tumor without any evidence of toxicity. In contrast, oral treatment at a dose of 800mg/kg/d exhibited little efficacy whereas severe toxicity existed if the dosage is higher. Collectively, intravesical metformin displays potent inhibition on bladder cancer *in vitro* and this preclinical study reveals the profound therapeutic application of metformin with durable tolerance via intravesical administration route.

## INTRODUCTION

Bladder cancer is one of the most frequent cancers of the urinary tract, accounting for about 74,000 new cases and 16,000 deaths in the United States in 2015[1]. Even though transurethral resection has served as the standard treatment, recurrence and metastasis are often seen in clinic [2]. The commonest way to prevent recurrence and progression is supplemented with intravesical chemotherapy or immunosuppressive agents [3–4]. However, these methods are largely restricted with various degrees of side effects such as bone marrow suppression, allergic reactions and etc[5]. Thus, there is an unmet demand to discover safe and effective drugs for treating bladder cancer.

Abnormality of metabolism is one typical characteristic of tumors proposed in Warburg effect. There is renewed interest in developing novel anti-cancer breakthroughs modulating metabolism to limit neoplastic growth. Tumorigenesis is a multistep process and tumor cells often display alterations in their energy metabolism to support rapid growth [6]. Metabolic reprogramming might be a profound strategy for cancer treatment. Metformin, a widely prescribed drug for treating type II diabetes, is one of the most extensively recognized metabolism modulators. Epidemiological evidence indicates that metformin has a high potential efficacy as an anti-cancer drug [7–10]. Data collected from various xenograft cancer models suggest that metformin delays the progression and relapse of cancer but no much research in bladder cancer is available [11–12].

Moreover, over two hundreds of clinical trials on anticancer studies have either been completed or are on going. However, no positive clinical results have been reported yet. Concentration of metformin on target organs is probably the major obstacle for these unsatisfactory results [13]. We notice that oral administration is the general treatment route in these bench work studies and clinical trials. The maximum concentration of metformin in circulating system is less than 60 μM via oral administration, far less away from 2 mM, the minimum effective concentration *in vitro* [14]. Moreover, increase of oral dosage of metformin causes the risk of lactic acidosis, having a vicious circle with the lactic acidosis produced by tumors via anaerobic glycolysis. Therefore, alternative administration to achieve an effective dose is critical [13].

Thus, in this present study, we aim to explore the efficacy of metformin using intravesical administration to treat bladder cancer. To achieve this aim, MB49, the popular murine bladder cancer cell line, was applied to establish syngeneic orthotopic model. This study provides an effective strategy to eradicate bladder cancer.

## RESULTS

### Metformin inhibits bladder cancer cell proliferation

**Figure 1:**
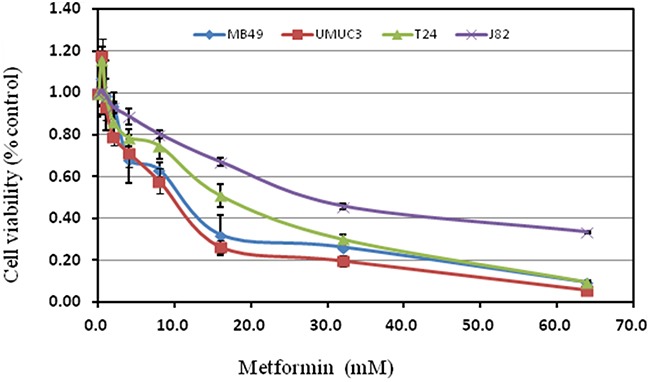
Treatment with metformin on cell proliferation of various bladder cancer cell lines Cell viability was assessed with 48 hour metformin treatment at concentrations ranging from 0 to 64mM on human bladder cancer cell lines T24, UMUC3, J82 and murine bladder cancer cell line MB49 using a tetrazolium-based assay. Results are presented as the median of 5 independent experiments.

These cell lines were exposed to 0. 5∼64 mM metformin. Interestingly, metformin generally promoted cell growth at less than 0.5mM concentration, then turned to a dose-dependent inhibition of cell proliferation. The most sensitive cell line is UMUC3 with IC_50_ 8.25mM while the most resistant one is J82 (IC_50_30.24mM) (Figure 1, Table 1).

**Table 1:** Inhibitory concentration 50%(IC_50_) for metformin

Metformin(mM)	Bladder cancer cell lines				
	MB49	UMUC3	T24	J82
	10.41	8.25	14.25	30.24

### Metformin suppresses colony formation

**Figure 2:**
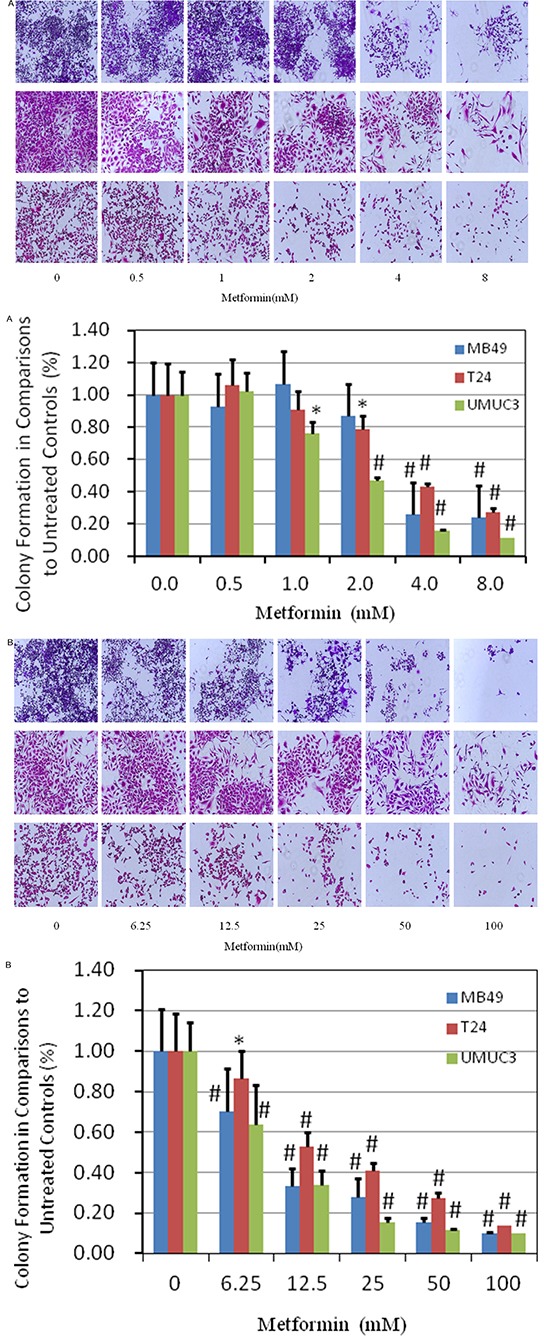
Evaluation of colony suppression of metformin on bladder cancer cell lines **A.** clonogenic assay was assessed after 7 day metformin treatment at various concentrations and stained with crystal violet at the end of the experiment. Above: images were taken through an inverted microscope with ×10 magnification. Below: the quantification of colony was determined by microplate area scan at OD 550nm, Results are presented as the median of 5 independent experiments (*P<0.05, ^#^*P*<0.01). **B.** colony formation assay was carried out with two hour treatment at labeled concentrations, twice per week for two weeks and stained with crystal violet at the end of the experiment. Above: images were taken through an inverted microscope with ×10 magnification. Below: the quantification of colony was determined by microplate area scan at OD 550nm, Results are presented as the median of 5 independent experiments (*P<0.05, ^#^*P*<0.01).

Colony formation was examined in the presence of metformin with either regular continuous or intermittent fashion. In regular continuous fashion, it was found that 0.5mM metformin favored the colony formation as shown in Figure 2A, Supplementary Figure S1. However, suppressive effect increased when the concentration of metformin was higher than 2mM in T24 and UMUC3 cell lines. Inhibitory effect in J82 was not significant, showing similar pattern observed in proliferation assay described above.

To mimic intravesical treatment, we designed an intermittent treatment protocol with 2 hour metformin incubation twice per week for two weeks (Figure 2B, Supplementary Figure S2). Understandably, much higher concentration of metformin is needed for inhibiting the colony formation, compared with that in continuous fashion in general (Figure 2B, Supplementary Figure S2). MB49 and UMUC3 were more sensitive than T24, same trend observed in continuous fashion. The dramatic suppression on colony formation of intermittent treatment imply that intravesical treatment is applicable to cause significant cell killing effects.

### Metformin activates AMPK

**Figure 3:**
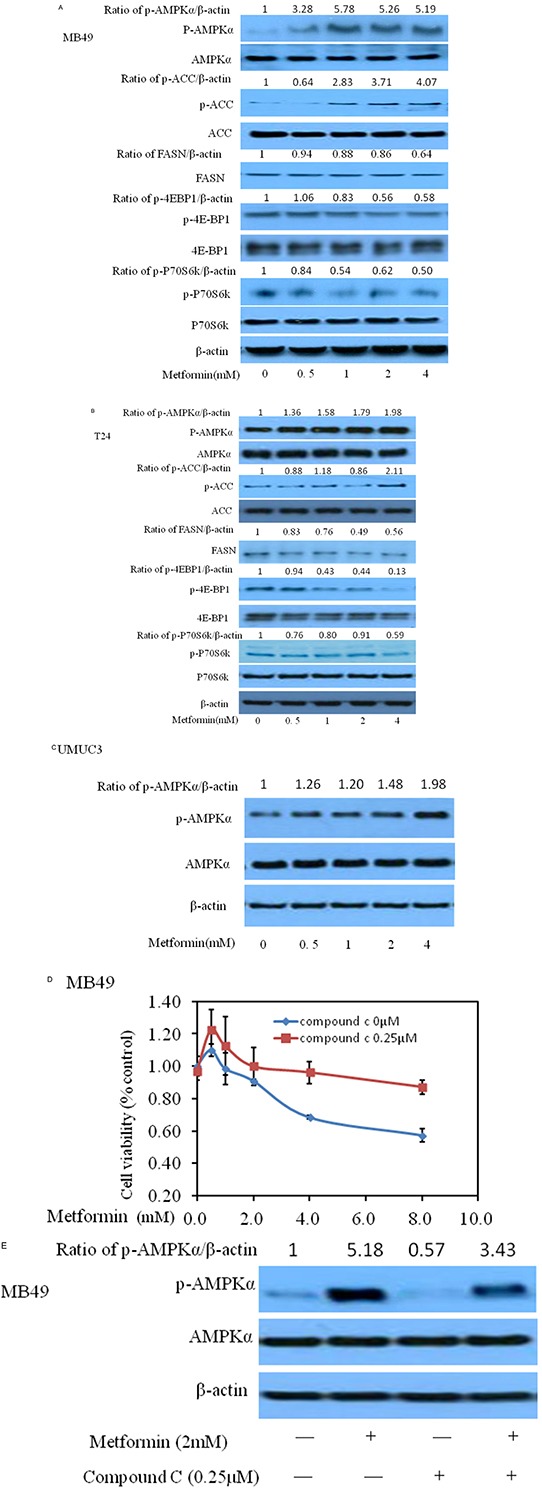
Effects of metformin on AMPK intracellular signaling pathways in three bladder cancer cell lines MB49, T24, and UMUC3 Panels **A–C** represent western blottings of p-AMPK, p-ACC, FASN, p-p70S6K, p-4EBP1, t-AMPK, t-ACC, t-p70S6K and t-4EBP1 of three bladder cancer cell lines MB49, T24, and UMUC3, respectively. β-actin was included as a loading control. The ratio of different proteins to β-actin was calculated by the band density of Western blots of each cell line using Image J software. **D**. Cell viability was assessed with 48 hour metformin alone or metformin combined with 0.25μM AMPK antagonist compound C treatment. Compound C significantly reduced the decrease of cell proliferation caused by metformin. **E.** Western blotting of p-AMPK and t-AMPK after treatment of either metformin or/and compound C.

AMPK, a central energy sensor, is known as a crucial factor in the interaction between metabolism and cancer [15]. As the activation of AMPK correlates tightly with phosphorylation at Thr-172 (p-AMPKα), we assessed activation of AMPK by determining phosphorylation AMPKα and its primary downstream targeting enzyme such as p-p70s6k, p-4EBP1 and p-ACC. As shown in Figure 3A–3C, metformin activated AMPK markedly and decreased the phosphorylation of downstream proteins including 4EBP1 and P70S6K. In the meantime, metformin increases the protein level of p-ACC then inhibits FASN, its downstream of AMPK-ACC, consistent with previous reports [16–17].

Next, we added AMPK specific inhibitor compound C to determine whether activating AMPK signaling pathway is essential for metformin to exert anti-tumor effect. As shown in Figure 3D, the ability of metformin to inhibit MB49 cancer cell proliferation was reduced after adding compound C, implying that inhibition of AMPK accelerates proliferation of cancer cells, consistent with western blot results (Figure 3E). Similar trends of human bladder cancer cells T24 and UMUC3 were observed (Supplementary Figures S3 & S4).

### Both AKT and ERK signaling pathways participate in inhibitory effect of metformin on bladder cancer cell growth

**Figure 4:**
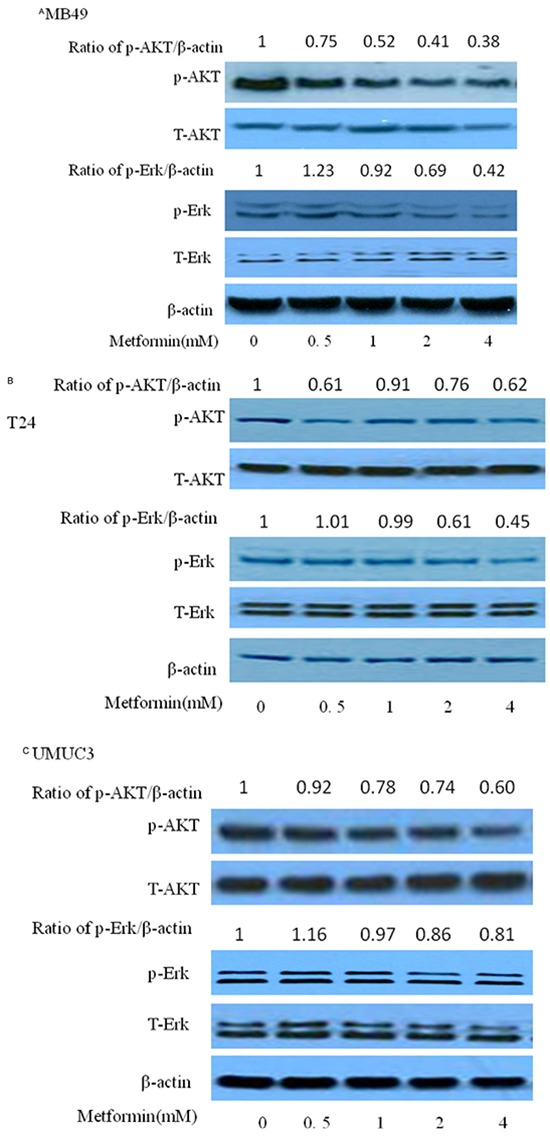
Effects of metformin on AKT and ERK in three bladder cancer cell lines MB49, T24, and UMUC3 Western blotting of p-AKT, p-ERK, t-AKT and t-ERK of MB49 **A.** T24 **B.** and UMUC3 **C.**, respectively. β-actin was included as a loading control.

**Figure 5:**
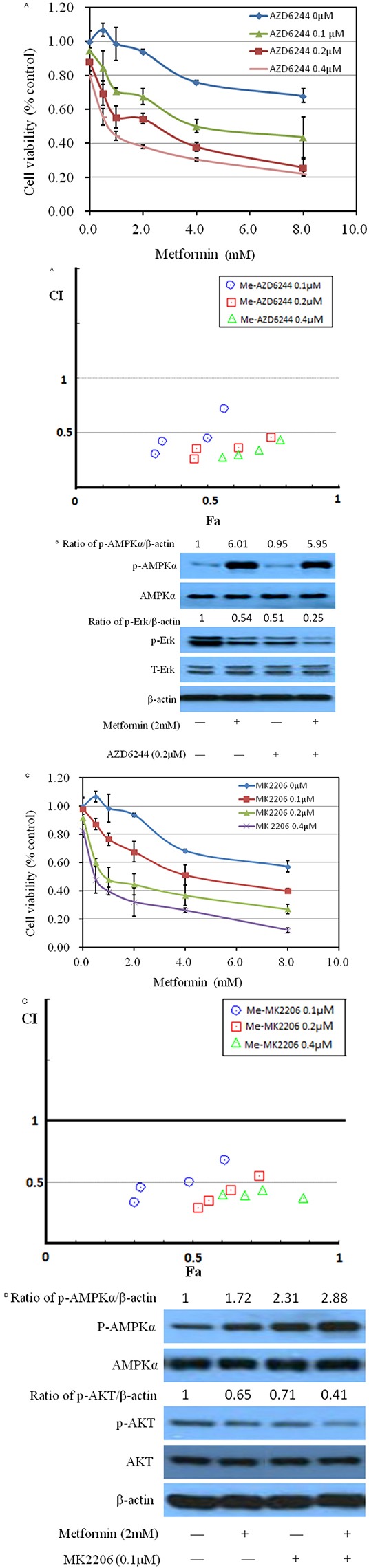
Effects of metformin on MB49 combined with ERK or AKT specific drugs **A.** Metformin combined with ERK specific inhibitor AZD6244 inhibited MB49 proliferation synergistically. Above: Cell viability was assessed with 48 hour metformin at 0, 1, 2, 4, 8mM alone or metformin combined with various concentrations (0.1, 0.2, 0.4μM) of AZD6244 treatment. Below: Combination index (CI) among the combinations of two drugs were calculated using CompuSyn software. If CI = 1, it denotes additivity; if CI > 1, it denotes antagonism; if CI < 1, it denotes synergism. CI values in the vast majority of combinations weres less than 0.5, indicating moderately strong synergism. **B.** Western blotting of p-ERK, t-ERK, p-AMPK and t-AMPK following treatment with metformin at 2mM and AZD6244 at 0.2μM in MB49. β-actin was included as a loading control. **C.** Metformin combined with AKT specific inhibitor MK2206 inhibited MB49 proliferation synergistically. Cell viability was assessed with 48 hour metformin at 0, 1, 2, 4, 8mM alone or metformin combined with indicated concentrations of MK2206 treatment. Combination index (CI) were calculated. **D.** Western blotting of p-AKT, p-AMPK, t-AKT and t-AMPK following treatment with the indicated concentration of metformin and AZD6244 in MB49. β-actin was included as a loading control.

Phosphorylated AKT and ERK are key intracellular mediators of cell survival and proliferation signals. Recent findings have confirmed that activated mutation of PI3k is associated with improved recurrence-free survival [18–20]. Thus, inhibition of Akt, the downstream of PI3K pathway, would be beneficial to block cancer cell growth [21–22]. Examining these molecular pathways during metformin treatment is meaningful for us to elaborate its anticancer mechanism. As shown in Figure 4A–4C, metformin decreased the phosphorylation of Akt and/or Erk1/2 at higher than 0.5 mM with no significant change on the protein levels of total Erk1/2 and AKT, in MB49, T24 and UMUC3, indicating that the inhibitory effect of metformin is correlated to the PI3K and Erk signaling pathways. A slight increase of phosphorylation of Erk1/2 at 0.5 mM in MB49 and UMUC3 cells is associated with the results of increase of colony formation at this concentration in Figure 2A. There was no significant difference of these signaling pathways among these cell lines. These data prompted us to further probe the role of Erk1/2 and AKT pathway by these inhibitors. As shown in Figure 5A and 5C, either AZD6244, the inhibitor of Erk1/2, or MK2206, the inhibitor of AKT exerts synergistic inhibition of cancer cell growth with metformin respectively, in a dose dependent fashion. This indicated that both Erk1/2 and AKT pathways got involved in events of inhibition of metformin. Interestingly, western blot results demonstrate that MK2206 alone increases protein level of phospho-AMPK while AZD6244 does not, indicating that inhibition of Akt may exert a different effect on phospho-AMPK, compared to inhibition of Erk, which warrants further investigation (Figure 5B and 5D). However, both MK2206 and AZD6244 amplify metformin-induced phosphorylation of AMPK (Figure 5B and 5D). Inhibition of either AKT or Erk1/2 will produce synergistical effect with metformin but MK2206 and AZD6244 may exert these effects via different molecular ways which also warrant further investigation.

### Intravesical rather than oral administration of metformin exert potent anti-cancer effects on orthotopic bladder tumors

Orthotopic mouse model was established to provide the useful tool to determine the effect of intravesical localized treatment [23]. Syngeinic tumor implantation provided better tumor take rate compared to the xenograft implantation [24].

Further, experimental trials on cancer cell implantation were conducted with harder but safe scratching on bladder wall to guarantee 100% tumor take rate. After achieving this rate, we designed intravesical and oral administrations, comparing efficacy and toxicity between the two different routes.

**Figure 6:**
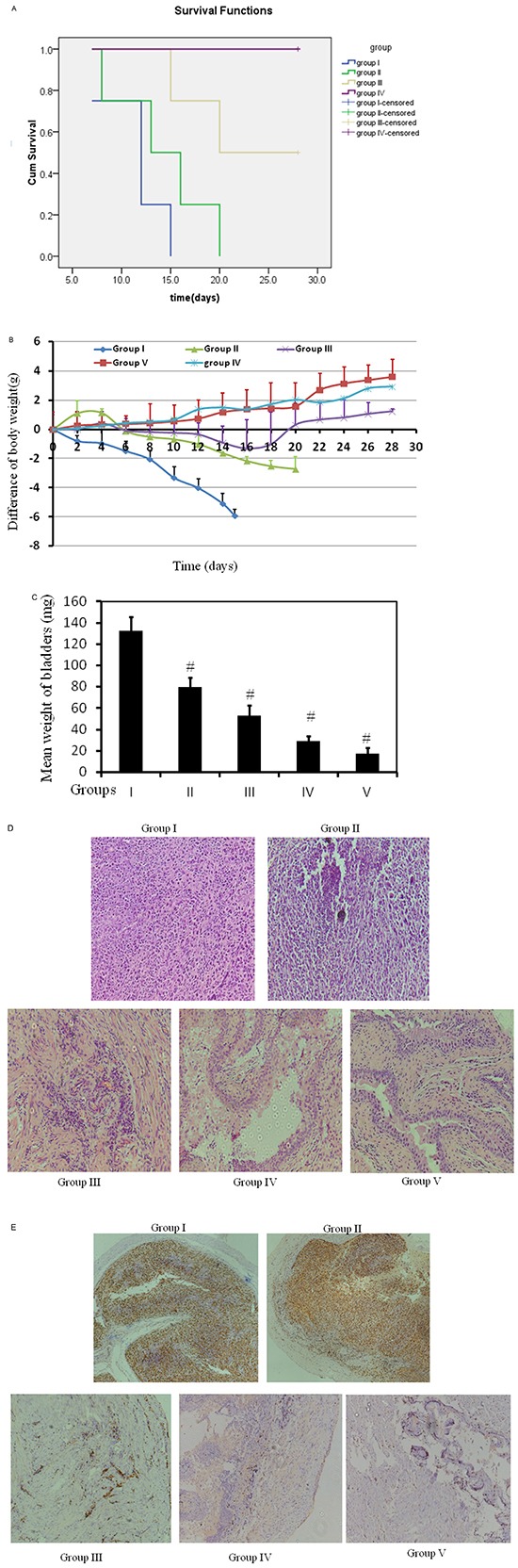
Bladders of female C57/BL6 mice were orthotopically implanted with MB49 cells (1.2×105) and divided into 4 groups randomly (*n* = 12 each group) Orthotopic bladder cancer implantation was processed for mice in groups I, II, III, and IV. Group V was mice without treatment without cancer cell implantation. Groups I and II were treated with either PBS or 800mg/kg/d metformin in PBS orally. Oral treatment started at day 2 and treated daily. Groups III and IV were intravesically treated with 50 μl either 80mM or 320mM metformin, respectively in PBS per mouse, starting at day 2, twice per week for two weeks. **A.** Kaplan-Meier survival analysis of five groups. Death of mice was checked daily and cumulative survival rate was plotted against the time course. Life span was 12.5 days vs 16 days in groups I and II mice, respectively. The weight of mice was measured daily **B.** and all living mice were sacrificed at day 28. **C.** Weights of mouse bladders including those died before the end of experiment were measured. All bladder tissues were collected and fixed. Histological sections from these tissues were subjected to H&E stain or immunohistochemistry for Ki67 to confirm the presence or absence of tumors **D.** & **E.**

In total, five groups were designed, 2 control groups: I-control with tumor and V- control without tumor, and 3 treatment groups: II- oral-treated group (800mg/kg/d), III- intravesical treated group (80mM, 50μL, equivalent to 26 mg/kg, twice per week), and IV- intravesical treated group (320mM, 50μL, equivalent to 104 mg/kg, twice per week). All treatments started at day 2 post tumor implantation for two weeks. Orthotopic bladder cancer implantation was processed for mice in Groups I, II, III, and IV except Group V, control mice without treatment in the absence of tumor. Figure 6A shows cumulative survival curves of five groups. Cancer cell implantation induced death of mice (Group I) but intravesical treatment dramatically enhanced life span with better survival in higher dose (Group IV vs Group I, *p*<0.001). Identical cumulative survival curves were observed in Groups IV and V, suggesting a potent anticancer efficiency of high dose of intravesical metformin. Oral treatment exerts very mild benefit in improving survival (Group II vs Group I, *p* = 0.015). Furthermore, there is a close correlation between the decrease of mouse body weight and the progression of tumor, based on observations of tumor implantation trials (data not shown). Therefore, decrease of mouse body weight could be a surrogate for tumor progression. As shown in Figure 6B, the decrease of body weight in Group I is seen, indicating the toxicity induced by tumor implantation. This decrease in body weight is attenuated by oral metformin at dose of 800mg/kg/d (Group II). However, oral administration is not able to reverse this decrease. In contrast, intravesical treatments at both doses reverse this decrease with better efficiency at higher dose (320mM). Furthermore, at the end of experiment, the weight of bladders was examined. The weight of every bladder in Group I was much bigger than that in Group V, indicating 100% existence of tumors in all mice in Group I (Figure 6C). Intravesical treatment profoundly diminished bladder weights at both doses. The bladder weights with higher dose are almost back to normal (Group IV vs Group V, *p* = 0.081). Oral administration reduced the bladder weights as well but this reduction was the mildest (132.8 mg vs 80.1 mg, Figure 6C). Furthermore, H&E results show the complete absence of tumor in Group IV (Figure 6D). In contrast, oral administration (Group II) causes much more residue of tumor. Ki67 staining demonstrates similar efficiency pattern Figure 6E. In all, intravesical treatment exhibited potent anti-cancer effect.

### Lactic acid and toxicity

**Figure 7:**
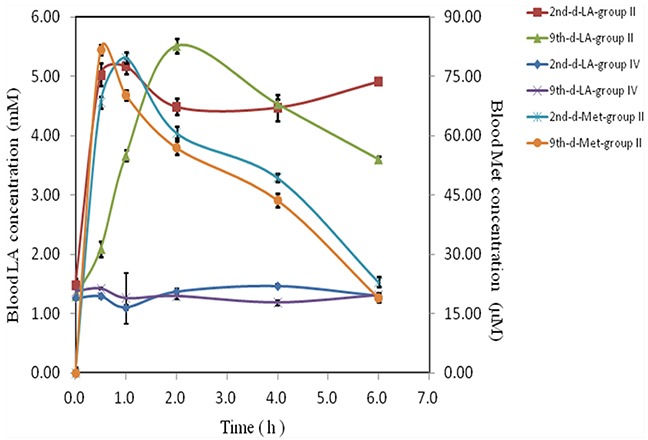
Toxicity of metformin Nausea, hematuria, vomiting or other gastrointestinal reactions of mice with various treatments were recorded. Blood samples from oral and high dose intravesical treatment groups (Groups II an IV) at various time points (0-6 hours) on either day 2 or day 9, were collected, lactic acid (LA) and metformin (Met) were measured (*n* = 12 per group).

Previous report has shown that the oral metformin in mice was 200mg/ml in drinking water [25], approximately equivalent to around 1000 mg/kg/d with gavage administration. To detect the toxicity of oral treatment, 1600mg/kg/d in mice was applied to determine the tolerance of mice. However, mice died successively at this dose with acute toxicity and behaved severe gastrointestinal side effects (data not shown). In contrast, neither visual vomiting, gastrointestinal side effects nor hematuria were observed in 800 mg/kg/d oral and two intravesical groups (II, III and IV) although hematuria is seen in Group I. Lactic acid and metformin in blood samples increased markedly in 800 mg/kg/d oral treatment (Group II) with maximum increase within two hours on day 2 and 9 respectively. Amount of lactic acid maintained within 7 hours whereas metformin disappeared sharply (Figure 7). In contrast, lactic acid in Group IV does not increase (Figure 7) and metformin is not detectable (data not shown), indicating that intravesical administration does neither increase any lactic acid nor any metformin leak into blood circulating system.

## DISCUSSION

In this study, we report pre-clinical results of intravesical treatment using a syngeneic orthotopic bladder cancer model and the molecular mechanism of mode of action is elaborated.

Intravesical treatment of metformin showed a rapid elimination of the implanted tumor twice per week after 2 weeks without any evidence of toxicity. Furthermore, mice are very tolerated with these doses. Interestingly, our *in vitro* data show that metformin at 0.5 mM enhances rather than blocks colony formation (Figure 2A), indicating that the sufficient dose is essential to exhibit anticancer effect. A recent negative report of oral metformin on pancreatic cancer from clinical trial strongly highlights the importance of dose concentration on target organ [26]. At the other hand, increasing oral metformin does not help to enhance its blood concentration rather than generating more side effect due to pharmacokinetics of metformin [27]. Higher dose of oral metformin arise gastrointestinal discomfort and lactic acidosis [28], as evidenced in this study as well. Markedly, intravesical administration is able to overcome these obstacles and exhibit potent effects.

The basis of molecular anticancer mechanism of metformin is activation of AMPK, proven by previous reports [29]. Interestingly, metformin is able to increase the longevity which is related to AMPK signaling pathway as well [30–33]. Another new proposal is that metformin functions primarily as antiforlate agent and secondarily as AMK activator [34]. We present new findings that AKT and ERK signaling participates in this event.

In summary, the intravesical administration of metformin yields a high rate of tumor clearance after 2 weeks therapy. In addition, intravesical treatment limits systemic exposure and reduces non-specific toxicities. This study has demonstrated the promising result of anti-bladder cancer effects in orthotopic preclinical animal model. This will encourage us to further explore its clinical application and/or combine metformin with other chemotherapy.

## MATERIALS AND METHODS

### Reagents

Metformin was purchased from Aladdin chemistry Co.Ltd and was diluted across a range of concentrations in culture media. Antibodies against total Akt, phosphor-Akt(Ser473), total Erk1/2, phosphor-Erk1/2, phosphor-Acetyl-CoA Carboxylase (Ser79), total Acetyl-CoA Carboxylase (Ser79), total p70 S6 kinase (Thr389), phosphor-p70 S6 kinase (Thr389), total AMPKα (Thr172), phosphor-AMPKα (Thr172), total 4E-BP1, phosphor-4EBP1, FASN and β actin were purchased from Cell Signaling Technology. AZD6244 and MK-2206 were from Selleckchem (Houston, Texas, USA).

### Cell lines and culture conditions

Murine and human bladder cancer cell lines provided by Dr. P Guo were cultured in DMEM supplemented (Hyclone, Logan, UT, USA) with 10% of FBS (Hyclone, Logan, UT, USA) and 1% of penicillin-streptomycin at 37°C, in humidified air containing 5% of CO_2_.

### Cell viability and cologenic assay

Cell viability was assessed using a tetrazolium-based assay using microplate reader (Biotek, SYNERGY HTX, Vermont, USA). IC_50_ values were determined through the dose-response curves.

Cologenic survival was defined as the ability of the cells to form colonies. Images were taken and analyzed by microscopy (Leica, DFC450C, Wetzlar, Germany) and microplate reader (Biotek, SYNERGY HTX, Vermont, USA).

### Protein characterization

Western blot assessment was performed using regular procedure in previous work [23]. Primary antibody was added in BSA and allowed to incubate overnight at 4°C, then washed with TBS/0.05% Tween-20 before secondary antibody was added and incubated for an additional hour at room temperature. The membrane was again washed 3 times before adding Pierce Super Signal chemiluminescent substrate (Rockford, IL, USA) and then immediately imaged on Chemi Doc (Bio-Rad, Hercules, CA, USA). The films were scanned using EPSON PERFECTION V500 PHOTO and quantified by Image J (NIH, Bethesda, MD, USA).

### Animals

Female C57BL/6 mice were purchased from Hunan SJA Laboratory Animal Co., Ltd (Changsha, Hunan, China). Animals were housed 4 per cage in a specific pathogen-free animal facility. The experimental protocol was reviewed and approved by the Institutional Animal Care and Use Committee at Hunan Normal University.

### Orthotopic implantation and intravesical treatment

Exponential growth of MB49 cells [35] were harvested and cell density in collection tube was counted by cell counter. Female mice 6 to 8 weeks of age were used for cancer cell implantation. The entire procedure of orthotopic implantation and intravesical treatment was similar to the previous published work [23] except that catheter scratching is much harder to guarantee 100% tumor cell implantation rate.

### Histologic analysis

Tissue processing, H&E and Ki67 staining of 7-μm tissue sections were conducted by Department of Pathology, Hunan Provincial Cancer Hospital, Changsha, Hunan, P.R. China. The slides were reviewed by a pathologist, Lei Xue. The pathology evaluation was done to confirm the presence or absence of tumor.

### Metformin and lactic acid measurement

Blood samples of mice were collected using the process described previously [36] from 0.5 to 6h after metformin was given via oral gavage. Blood lactic acid levels were measured by lactic acid assay kit (Sigma-Aldrich) and metformin concentrations were determined by HPLC, as reported previously [37].

### Statistical analysis

All data are presented as mean±SEM. Statistical analyses were carried out using ANOVA analysis and statistical significance was assumed at a value of *p*<0.05.

## SUPPLEMENTARY FIGURES


