# Supplementary material for: High efficacy of intravesical treatment of metformin on bladder cancer in preclinical model
Source: Oncotarget. 2016 Jan 18;7(8):9102–17. doi: 10.18632/oncotarget.6933 (PMC4891029; doi:10.18632/oncotarget.6933)
Supplement: Supplementary file 1 [file oncotarget-07-9102-s001.pdf]

SUPPLEMENTARY FIGURES

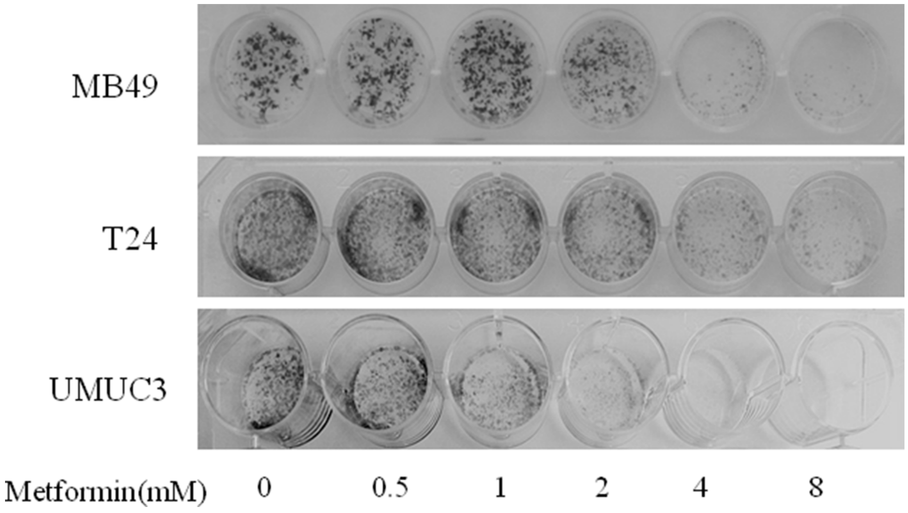

Supplementary Figure S1: Evaluation of colony suppression of metformin on bladder cancer cell lines. clonogenic assay was assessed after 7 day metformin treatment at various concentrations and stained with crystal violet at the end of the experiment. Photograph was taken through high resolution camera. Results are presented as the median of 5 independent experiments.

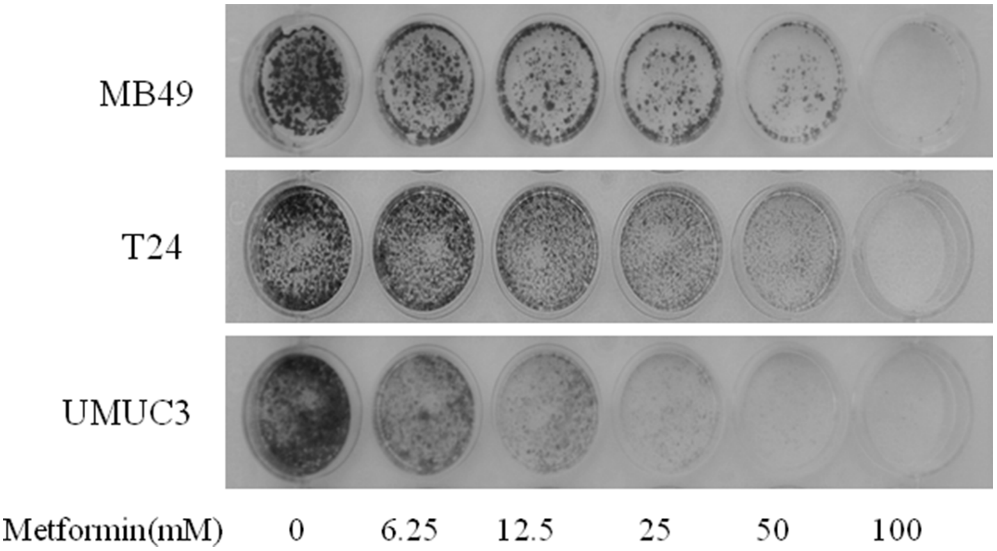

Supplementary Figure S2: Imitating intravesical evaluation of colony suppression of metformin on bladder cancer cell lines. Colony formation assay was carried out with two hour treatment at labeled concentrations, twice per week for two weeks and stained with crystal violet at the end of the experiment. Photograph was taken through high resolution camera. Results are presented as the median of 5 independent experiments.

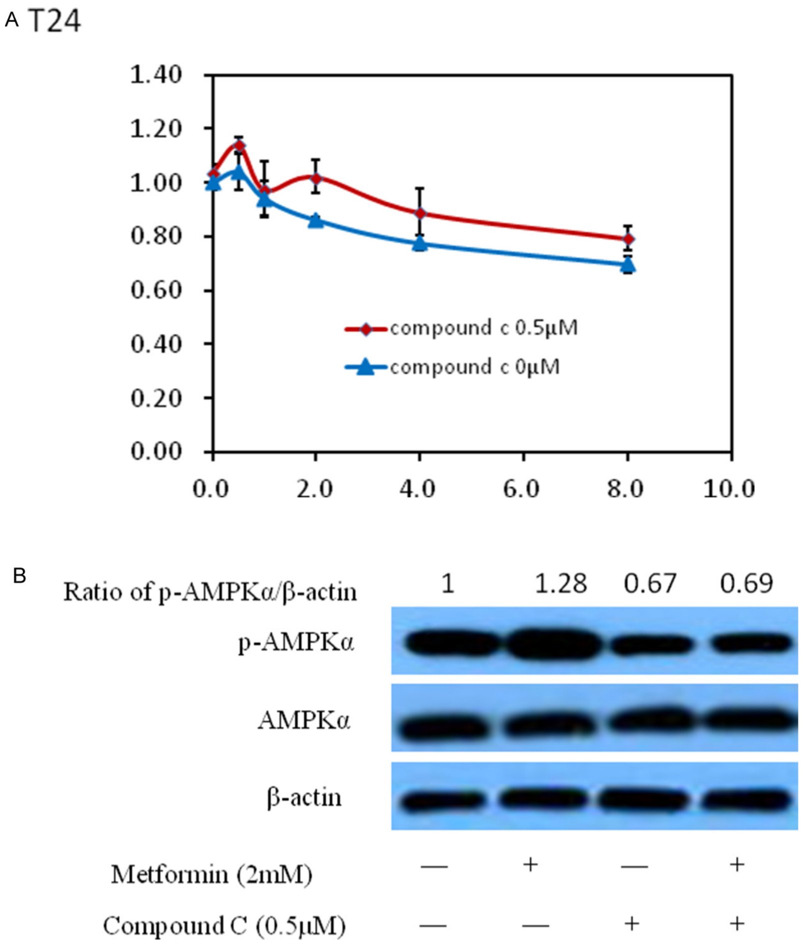

Supplementary Figure S3: Influence of AMPK specific inhibitor compound C on the action of metformin in human bladder cancer cell line T24. A. Cell viability was assessed with the treatment of 48 hour metformin alone or combined with 0.5 μM compound C. Compound C reduced the decrease of cell proliferation caused by metformin. **B.** Western blotting of p-AMPK and t-AMPK after treatment of either metformin or/and compound C. β-actin was served as a loading control.

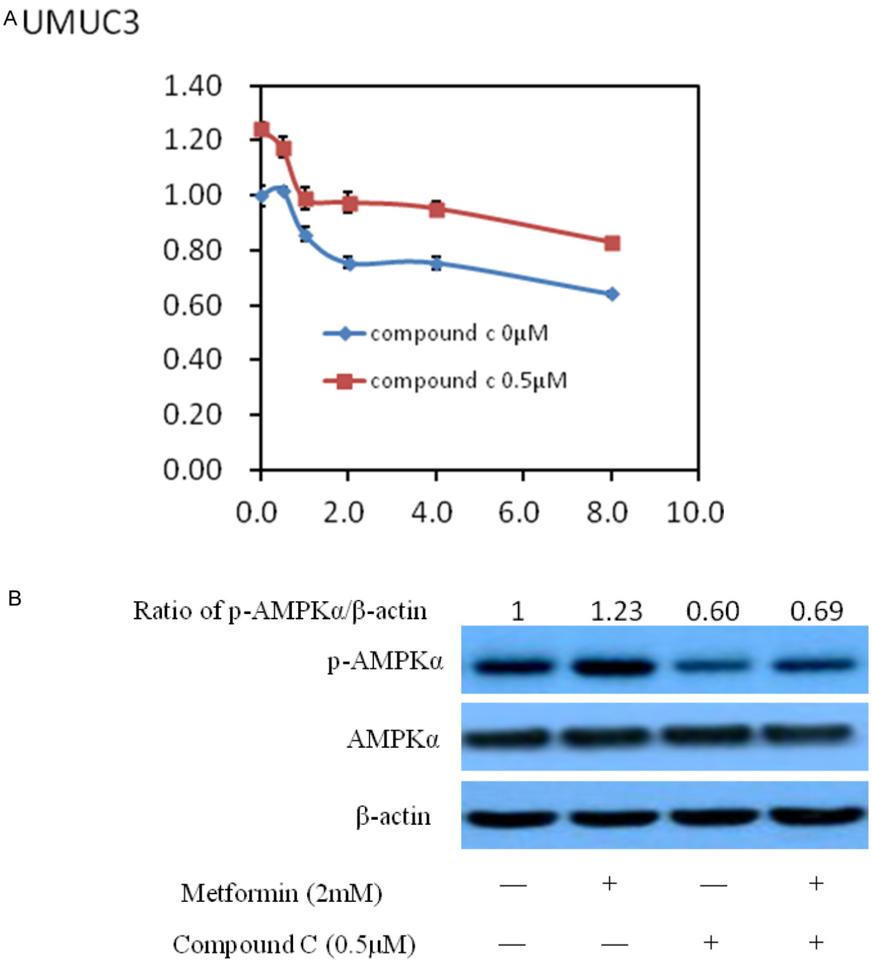

Supplementary Figure S4: Influence of AMPK specific inhibitor compound C on the action of metformin in human bladder cancer cell line UMUC3. A. Cell viability was assessed with the treatment of 48 hour metformin alone or combined with 0.5 μM compound C. Compound C reduced the decrease of cell proliferation caused by metformin. **B.** Western blotting of p-AMPK and t-AMPK after treatment of either metformin or/and compound C. β-actin was served as a loading control.
